# Exome sequencing of patients with syndromic tall stature reveals four novel candidate genes

**DOI:** 10.1530/EC-25-0137

**Published:** 2025-07-15

**Authors:** Gabriela Jeesoo Kim, Edoarda Vasco de Albuquerque Albuquerque, Raissa C Rezende, Laurana De Polli Cellin, Ana Maria Santillan Vasconez, Ana C V Krepischi, Lucas Santana, Antônio Marcondes Lerario, Vinicius de Souza, Renata Scalco, Alexander A L Jorge

**Affiliations:** ^1^ Unit of Genetic Endocrinology (LIM25), Hospital das Clínicas da Faculdade de Medicina da Universidade de São Paulo, São Paulo, Brazil; ^2^ University of Michigan Medical School, Ann Arbor, Michigan, USA; ^3^ Unit of Clinical Medicine, Hospital Universitário Professor Alberto Antunes, Maceió, Brazil; ^4^ Department of Genetics and Evolutionary Biology, Institute of Biosciences, Universidade de São Paulo, São Paulo, Brazil; ^5^ Department of Internal Medicine, Division of Metabolism, Endocrinology and Diabetes, University of Michigan, Ann Arbor, USA

**Keywords:** tall stature, exome sequencing, candidate gene, syndromic tall stature, copy number variant, variant

## Abstract

This study aimed to evaluate a cohort of patients with syndromic tall stature of unknown etiology via exome sequencing (ES) to identify novel candidate genes for overgrowth conditions. We enrolled 37 patients with heights greater than the 97.7th percentile and syndromic features. The ES analysis first focused on rare deleterious single nucleotide and copy number variants in genes previously associated with overgrowth conditions. For patients in whom diagnosis of a known tall stature disorder could not be achieved, we performed analysis for candidate genes. The search considered deleterious variants in constrained genes that were linked with height in association studies, animal models consistent with the proposed phenotype, and/or variants recurrent in the literature or our cohort. Genetic diagnosis was established in 11 patients. Pathogenic or likely pathogenic variants were identified in *FBN1*, *PTEN*, *NSD1*, *SUZ12*, *CDH8*, and *DEPDC5*. One patient carried a likely pathogenic mutation in *FBN2* and a pathogenic mutation in *COL5A1*. Furthermore, in two patients, we identified large pathogenic deletions confirmed by chromosomal microarray analysis. Candidate gene analysis uncovered four genes potentially associated with tall stature in five patients: *PTCH1*, *SST*, *KDM4A*, and *GRB10*. *PTCH1* and *SST* were identified in patients with whole gene deletions. Two unrelated patients were found to have the same rare missense variant in *KDM4A*. In conclusion, exome sequencing analysis had a diagnostic yield of 29.7% in a cohort of patients with syndromic tall stature and we identified four novel candidate genes that are involved in overgrowth conditions.

## Introduction

Tall stature is defined as a height greater than the 97.7th percentile or two standard deviations (SDS) above the mean for a population of individuals of the same age, sex, and ethnicity (1). Most cases of tall stature are due to familial tall stature, also known as constitutional tall stature (2). Familial tall stature is usually defined as a child’s height aligning with mid-parental height without dysmorphic or syndromic features or body disproportionality (3). When a child has familial tall stature, their height is presumed to be due to polygenic causes.

In contrast, syndromic tall stature is generally due to a mutation in one of the critical genes for the control of growth (4). Monogenic causes of syndromic tall stature result in a recognizable pattern of clinical characteristics that include dysmorphisms, malformations, and/or neurodevelopmental disorders (1, 2). Clearly defined causes of syndromic tall stature include Sotos syndrome (OMIM 117550), Weaver syndrome (OMIM 277590), Simpson-Golabi-Behmel syndrome (OMIM 312870), Marfan syndrome (OMIM 154700), homocystinuria (OMIM 236200), Beckwith-Wiedemann syndrome (OMIM 130650), and Imagawa-Matsumoto syndrome (OMIM 618786) (2, 5, 6). Unfortunately, many patients with syndromic tall stature remain without a definitive diagnosis for their complex phenotype after clinical evaluation.

Thus far, few articles have been published regarding the diagnosis of syndromic tall stature by genetic testing. The diagnostic yield of these studies ranges from 32 to 49% (1, 3, 7), but there exists heterogeneity regarding the characteristics of the studied populations and the genetic testing techniques used.

Despite headways made in recent years regarding the accessibility of genetic testing and the discovery of new syndromes, barriers to diagnosis still exist. This may be partially explained by rare conditions involving new gene–disease associations that cause syndromic tall stature and have yet to be characterized.

In this study, we aimed to evaluate a cohort of patients with syndromic tall stature from a single tertiary center using exome sequencing (ES) analysis to both identify the genetic causes for their tall stature and uncover new candidate genes for tall stature in the event that genetic diagnosis could not be reached.

## Methods

### Patient selection

We enrolled 29 children and eight adults who were referred to the Pediatric Endocrinology outpatient clinic of a single tertiary academic center specialized in growth disorders for the evaluation of tall stature from January 2017 to February 2024.

Patients were included in the study if they had syndromic tall stature, if samples were provided for genetic testing, and if the genetic analysis was conducted via ES. Syndromic tall stature was defined as a height of two or more SDS above the mean for matched age and sex with the presence of additional characteristic features such as intellectual disability, developmental delay, major malformations, dysmorphic features, and/or congenital abnormalities affecting specific organ systems (1, 8). All patients underwent careful clinical assessment by specialized endocrinologists and/or geneticists. All female patients were confirmed to be 46,XX, and all male patients were confirmed to be 46,XY. Pituitary gigantism was also ruled out in all patients based on normal insulin-like growth factor 1 (IGF-1) levels and/or normal suppression of growth hormone (GH) with glucose tolerance testing. Patients were excluded if it was possible to establish a clinical diagnosis of a syndromic tall stature condition based on the clinical evaluation or if patients had a diagnosis of precocious puberty. Twenty-two of the 37 selected patients were described in previous studies, with a proportion of these patients having genetic diagnoses previously reported as well (1, 8, 9). The ES data of these patients were reanalyzed and combined with the data of 15 new patients to determine the diagnostic yield of ES for syndromic tall stature and to identify novel candidate genes for this condition.

### Exome sequencing and copy number variant analysis

DNA was extracted from peripheral blood leukocytes of patients and available family members. ES was performed for all patients as previously described (8, 10). Trio analysis was performed for four patients, and ES was performed for only the index case for the remaining patients. Copy number variation (CNV) duplications or deletions identified by ES were confirmed using chromosomal microarray analysis (CMA), through a combination of the comparative genomic hybridization array (CGHa using 180 K Agilent, USA) and single nucleotide polymorphism array (SNPa).

### Variant filtering and diagnosis

Variants were analyzed using the Franklin by Genoox platform (franklin.genoox.com). We initially evaluated the presence of variants in genes already associated with syndromic tall stature by applying a virtual panel. The strategy to evaluate these variants has previously been reported (8). In summary, variants with a minor allele frequency (MAF) of less than 0.01% in population databases gnomAD (11) and ABraOM (12) were selected if they were present in coding regions or canonical splicing sites. We further prioritized variants predicted to be loss-of-function (LoF), including premature stop-gains, frameshifts, and splice site variants. Missense variants were prioritized based on prediction to be deleterious by *in silico* tools, as well as prior classifications of variants as pathogenic in ClinVar (13) or in previous publications. *In silico* predictions were defined as deleterious if they had a REVEL (14) ≥0.64 for missense variants and dbscSNV (15) ≥0.70 or SpliceAI ≥0.22 (16) for splice-altering variants. Finally, we considered factors such as inheritance pattern compatibility, medical literature, and concordance between patient phenotype and disorder-specific characteristics. Variants were categorized as pathogenic (P), likely pathogenic (LP), variant of uncertain significance (VUS), likely benign (LB), or benign (B) according to ACMG/AMP criteria (17).

The analysis of CNVs was performed giving priority to rare variants containing protein-coding genes with probability of haploinsufficiency (pHaplo) score ≥0.9 for deletions or probability of triplosensitivity (pTriplo) score ≥0.9 for duplications. CNVs were classified based on the recommendations of the ACMG/ClinGen (18).

### Candidate gene selection

For patients whose diagnosis could not be achieved, analysis was performed for candidate genes, which are genes without known human phenotypes listed in the Online Mendelian Inheritance in Man (OMIM) database. To determine the suitability of the genes in which we found variants as candidate genes for syndromic tall stature, we evaluated their intolerance for specific mutations, GWAS (19) catalog associations with phenotypes such as height and body mass, feasible mechanism of disease based on gene function, case studies (if available) in the medical literature regarding mutations in our candidate genes, recurrence of rare variants in patients with similar phenotypes within our cohort, and animal models consistent with proposed phenotypes. Intolerance to genetic variants was determined via a loss-of-function observed/expected upper fraction (LOEUF) score of <0.6 (11), a probability of being loss-of-function intolerant (pLI) score ≥0.9 (20), or a missense Z-score >3 (21) calculated based on gnomAD v4.1.0.

For the two patients for whom diagnosis was achieved through CNV analysis, candidate genes were selected among those mapped within the CNV through the criteria described above. Deleted protein-coding genes were further examined for LOEUF and pLI scores, and similar factors such as inheritance pattern compatibility, medical literature, patient phenotype, and disorder-specific characteristics were considered, as above. Specifically, we looked at all individual protein-coding genes mapped to the CNV to determine whether any of these genes were associated with the phenotypes under study. We also compared detected CNVs with those listed in the DECIPHER (22) database as a way of further confirming CNV-disease phenotype compatibility.

## Results

### Diagnostic yield

Genetic diagnosis was achieved in 11 of the 37 included patients for a diagnostic yield of 29.7%. Pathogenic or LP variants in *FBN1* were identified in three patients, and P or LP variants in *NSD1*, *SUZ12*, *CDH8*, and *PTEN* were observed in one patient each. In addition, one patient carried significant variants in two genes: an LP variant in *FBN2* and a P variant in *COL5A1* (Table 1). ES analysis further identified two patients with pathogenic deletions confirmed by CMA (Table 2). Finally, one patient was found to have a P variant in *DEPDC5* that explained his neurodevelopmental phenotype but not his tall stature (Table 1).

**Table 1:** Genetic variants establishing patient diagnosis identified through ES analysis.

ID	Gene	Transcript	Variant	Protein	MAF	Clinvar	dbSNP ID	Segregation	Variant classification
1	*NSD1*	NM_022455.5	c.1610_1611del	p.Phe537Tyrfs*7	N/A	N/A	N/A	N/A	LP PVS1, PM2
2	*SUZ12*	NM_015355.4	c.1797A>C	p.Gln599His	N/A	N/A	rs1567840381	N/A	LP PM1, PM2, PP4_(moderate)_
3	*FBN2*	NM_001999.4	c.2783G>A	p.Trp928*	N/A	N/A	N/A	N/A	LP PVS1, PM2
3	*COL5A1*	NM_000093.5	c.3271G>T	p.Glu1091*	N/A	P (1)	N/A	N/A	P PVS1, PM2, PP5
4	*FBN1*	NM_000138.5	c.8227-2A>G	–	N/A	N/A	N/A	N/A	LP PVS1, PM2
5	*FBN1*	NM_000138.4	c.5072del	p.Arg1691Lysfs*24	N/A	N/A	N/A	N/A	LP PM1, PM2
6	*FBN1*	NM_000138.5	c.7039_7040delAT	p.Met2347Valfs*19	N/A	P (14), LP (1)	rs794728319	*de novo*	P PVS1, PS2, PM2
7	*CHD8*	NM_001170629	c.2154dupC	p.Phe719Leufs*2	N/A	LP (1)	N/A	N/A	LP PVS1, PM2
8	*PTEN*	NM_000314.8	c.512A>G	p.Gln171Arg	N/A	LP (1)	rs786204865	N/A	P PS3^†^ +PS1 + PM2+PP3-strong
9	*DEPDC5* ^‡^	NM_001242897.1	c.715C>T	p.Arg239*	N/A	P (7)	rs587776976	N/A	P PVS1, PM2, PS4

ES, exome sequencing; MAF, highest allele frequency observed in gnomAD database; P, pathogenic; LP, likely pathogenic; VUS, variant of uncertain significance; S, supportive. Cases 1, 2, and 7 were previously reported in (1). Cases 3 and 4 were previously reported in (8).

^†^ Evidence for PS3 comes from (51).

^‡^ This variant is responsible for the neurological sequelae of ID 9 only, not his tall stature.

**Table 2:** Chromosomal variants detected by ES and confirmed by chromosomal microarray analysis in patients with syndromic tall stature.

ID	CNV	Band	Genome build GRCh38		Size	Probable disease-causing gene	Segregation	ACMG variant classification
			Start point	End point				
13	Deletion	9q22.32	94443367	96223323	1.8 Mb	*PTCH1*	Non-maternal	Pathogenic (1A, 2A = 1.0)
14	Deletion	3q27.3q28	187407704	191030665	3.6 Mb	*SST*	*De novo*	Likely pathogenic (1A, 2A, 4C = 0.9)

ES, exome sequencing; CNV, copy number variation. Case 13 was previously reported in (1).

### Candidate genes

Two candidate genes were identified in single nucleotide variants (SNVs), and two were identified in CNVs in five patients (Table 3), with two unrelated patients sharing the same variant in *KMD4A* (kinship score −0.0695) (23). All variants were rare in public population databases. Variants included missense mutations in *GRB10* and *KDM4A* and whole gene deletions of *SST* and *PTCH1*, all identified as being heterozygous. Three of these genes are intolerant of LoF variants (*GRB10*, *KDM4A*, and *PTCH1*).

**Table 3:** Candidate genes for the phenotype of tall stature and the identified genetic variants.

Gene	Transcript	Variant	Protein	dbSNP ID	MAF (%)	In silico prediction (revel/dbscSNV)	Constraint metrics			
							pLI	LOEUF	Z	pHaplo
*GRB10*	NM_001350814.2	c.1685C>G	p.Thr562Ser	N/A	N/A	0.64	1	0.36	2.55	0.88
*KDM4A*	NM_014663.3	c.662G>A	p.Arg221His	rs1418933537	0.0056	0.69	1	0.156	−0.49	0.99
*SST*	N/A	Complete gene deletion		N/A	0	N/A	0.01	1.529	−0.39	0.90
*PTCH1*	N/A	Complete gene deletion		N/A	0	N/A	1	0.146	3.15	1.00

MAF, highest allele frequency observed in gnomAD database (for single nucleotide variants) or Database of Genomic Variants (for CNVs); pLI, probability of being loss-of-function intolerant score; LOEUF, loss of function observed/expected upper fraction score; Z, missense Z-score calculated based on gnomAD v4.1.0; pHaplo, probability of haploinsufficiency score.

Both missense variants (*GRB10* and *KDM4A*) have deleterious predictions (14, 24) (Table 3). *GRB10*, *SST*, and *PTCH1* are associated with increased body size in knockout (KO) animal models, and *SST* is additionally associated with increased levels of circulating GH (25, 26, 27, 28). All genes except for *KDM4A* were associated with body height in the GWAS catalog.

The 3q27.3q28 deletion was confirmed to be *de novo* via trio analysis. This segment contains 12 protein-coding genes, none of which have previously been associated with tall stature phenotypes. In the DECIPHER database, there are 39 individuals with available clinical data whose deletions overlap with the one observed in our patient. For four out of these 39 patients, tall stature is a characteristic reported phenotype. The minimal overlap of the genomic segments of these four CNVs and the CNV of our patient involves three genes: *RTP2*, *BCL6*, and *SST*, with the latter two genes being predicted to suffer haploinsufficiency (Supplementary Table 1 (see section on Supplementary materials given at the end of the article)).

The 9q22.32 deletion was confirmed to be absent in the patient’s mother and sister, but the patient’s father was unavailable for genetic analysis. The deletion involves seven protein-coding genes: *AOPEP*, *ERCC6L2*, *FANCC*, *FBP1*, *FBP2*, *MFSD14B*, and *PTCH1*; of these genes, only *PTCH1* is predicted to suffer haploinsufficiency (11). LoF variants in *PTCH1* are associated with basal cell nevus syndrome 1 (MIM #109400). Indeed, in addition to tall stature, this patient had a history of basal cell carcinoma and a neoplasm of the jaw. Twenty individuals with clinical data available are reported in the DECIPHER database as having deletions that overlap with that of our patient, three of whom are described as having tall stature. When considering the deletions of these three individuals, as well as the deletion of our patient, *PTCH1* is the only gene involved in all four deletions (Supplementary Table 1). Furthermore, there exists a fourth patient in the DECIPHER database with a point mutation involving *PTCH1*, who also presents with tall stature.

## Discussion

In many cases of patients with syndromic tall stature, it is highly likely that a rare variant in a single gene underlies their growth disorders. Despite the often monogenic nature of this condition, among studies investigating cohorts of patients with tall stature of unknown etiology, a definitive diagnosis has been established in fewer than half of the cases.

Tatton-Brown *et al.* analyzed 710 individuals with overgrowth, defined as height and/or head circumference ≥2 SD above the mean, accompanied by intellectual disability (7). Using a combination of targeted gene analysis and ES, they identified pathogenic variants in one of 14 genes in 347 individuals, resulting in a diagnostic yield of 49% (7). Similarly, Vasco de Albuquerque Albuquerque *et al.* employed karyotyping, targeted gene panels, CMA, and ES to diagnose 13 out of 30 (43%) syndromic tall stature patients with a definitive genetic condition (1). Gregorova *et al.* conducted another study involving 34 Czech patients with familial tall stature, both with and without additional dysmorphic features (3). Their approach – utilizing karyotyping, probe-dependent amplification for *SHOX* gene variants, and a targeted next-generation sequencing panel – yielded genetic diagnoses in 11 cases (32%) (3).

In our study, which analyzed a cohort of 37 patients using ES, we achieved a diagnostic yield of 29.7%. This relatively lower yield can be attributed to the exclusion of patients with clinical suspicion of conditions such as Marfan, Sotos, or Weaver syndromes, who were assessed using targeted panels (1). Consequently, the cohort likely included fewer individuals with a high probability of harboring pathogenic variants in genes already established as associated with tall stature. Nevertheless, patients with pathogenic variants in *FBN1* and *NSD1* were identified within the current cohort. In addition, one patient has a pathogenic variant in *DEPDC5* that explained his neurodevelopmental phenotype only (29); it is likely that another variant that has yet to be uncovered is what caused his observed tall stature.

The overall diagnostic yield of ES in the evaluation of syndromic tall stature aligns with that reported for patients with other syndromic growth disorders (10, 30) and isolated neurodevelopmental disorders (31). A subset of undiagnosed patients may harbor variants in genes not yet associated with human diseases, presenting a unique opportunity to uncover and characterize novel genetic syndromes. Within this framework, the present study identified four new candidate genes.

Of the four candidate genes for tall stature identified, there exists the most evidence in the literature for *GRB10* being associated with tall stature. Growth factor receptor-bound (GRB) 10 is a signaling adapter protein that binds to two tyrosine kinase receptors important in growth: the insulin-like growth factor type 1 receptor, the primary mediator of fetal growth, and the insulin receptor (32, 33). The *GRB10* variant identified in our patient (p.Thr562Ser) is predicted to disrupt the SH2 domain, which is critical for *GRB10* interaction with and inhibition of several tyrosine kinase receptors (34). There are various studies of mouse models demonstrating the relationship between *Grb10* and overgrowth both *in utero* and postnatally (25, 33). Interestingly, a recent study of *Grb10* KO mice showed that these mice developed a fetal overgrowth phenotype largely independent of the actions of insulin and IGF receptors (33). *GRB10* duplication has also been suggested as a genetic cause of Silver-Russell syndrome (MIM # 618905), a well-known short stature syndrome (35). More recently, a case study was published regarding a CNV deletion in chromosome 7, which involved several protein-coding genes including *GRB10* and resulted in a complex phenotype that included features of Beckwith-Wiedemann syndrome, a recognized prenatal overgrowth condition (36). Our patient, born large for gestational age, macrocephalic, and with tall stature (Table 4), provides further evidence that *GRB10* is likely the causative gene of an overgrowth syndrome.

**Table 4:** Clinical characteristics of patients with syndromic tall stature carrying candidate genes.

ID	Gene	Age (Y)	Birth weight SDS	Height SDS	Target height SDS	Head circumference SDS	Neurodevelopmental	Other clinical features
10	*GRB10*	5	+2.4	+2.0	−0.4	+2.3	ADHD, speech and language delay	Overweight, sparse hair, prominent forehead
11	*KDM4A*	12	−1.4	+3.3	−0.8	+1.9	Global developmental delay, intellectual disability	Hypotelorism, high palate, epilepsy, strabismus
12	*KDM4A*	16	N/A	+2.7	−0.2	+0.1	Learning difficulties (mild)	Hypertelorism, prominent antihelices, hallux valgus (bilateral), high palate, clinodactyly of the 5th toe (bilateral)
13	*PTCH1*	8	N/A	+2.7	−0.7	+5.6	Global developmental delay	Prominent forehead, concave nasal ridge, hypertrichosis, genu valgum, jaw neoplasm, basal cell carcinoma, twelfth rib hypoplasia, abnormality of the vertebral spinous processes, hypertrichosis
14	*SST*	21	+0.7	+4.5	+0.6	+3.4	ADHD	Absence seizures, mandibular prognathia, abnormal earlobe morphology, splenomegaly, esophagitis, gastritis (mild)

Y, years. N/A indicates the data were not available. Case 13 was previously reported in (1).

*KDM4A* (also known as *JMJD2A*) drew our attention as a candidate gene because the same variant was found in two unrelated patients with syndromic tall stature. *KDM4A*, which functions as a histone demethylase (37), is an epigenetic regulator of osteoblast and adipocyte differentiation and is involved in many other physiological processes such as the regeneration of skeletal muscle and embryonic stem cell differentiation (38, 39). There still is much to be elucidated about the exact mechanism by which KDM4A interferes with growth regulation. Prior studies have shown that inactivation of *KDM4A* can lead to inhibition of adipocyte formation and promotion of osteoblast differentiation (37). The variant identified in our patients (p.Arg221His) is located within the highly conserved catalytic domain, JmJC. A pathogenic mutation in the equivalent residue of *KDM4B* (p.Arg222Trp), a paralog of *KDM4A*, has previously been associated with an autosomal dominant intellectual developmental disorder (40). The two patients in our cohort with the same variant in *KDM4A* have varying levels of intellectual disability (Table 4). It is also notable that variants in genes involved in histone methylation have already been established as a known mechanism of disease in tall stature syndromes such as Sotos syndrome and Weaver syndrome (6, 41).

Among the genes contained within the two pathogenic CNV deletions identified, two candidate genes stood out. *PTCH1* encodes a receptor involved in the sonic hedgehog homolog (SHH) signaling pathway, which is important in the formation of embryonic structures and tumorigenesis, and functions to suppress SHH (42). *PTCH1* has also been found to be a negative regulator of Indian hedgehog (IHH) signaling in a mouse model of Brachydactyly type A1, a cause of short stature, with impaired interaction between IHH and receptor PTCH1 contributing to the phenotype (43). *PTCH1*, lost in the 9q22.32 deletion, is associated with basal cell syndromes in OMIM but is not yet associated with height. Previous reports suggest that CNVs of 9q22.3 can result in variations in height, with a prior description of a patient with overgrowth and macrocephaly due to a microdeletion of this region (44), as well as reports of short stature caused by microduplications of 9q22.32 (45). Our patient’s deletion involves seven protein-coding genes, but *PTCH1* is the only gene in the region that is predicted to suffer haploinsufficiency. In the DECIPHER database, there are four patients with pathogenic variants involving *PTCH1* (three microdeletions that overlap with that of our patient, and a splice site consensus variant), all of them with a tall stature phenotype (Supplementary Table 1). A case study describing a patient with a premature stop codon in *PTCH1* who had tall stature provides further evidence that *PTCH1* is the gene in this region responsible for alterations in height (46).

We were similarly able to identify *SST* as the candidate gene responsible for tall stature in the patient with the 3q27.3q28 deletion. Somatostatin (SST) is a neuropeptide that functions as an inhibitor of GH release (47). As far as we know, up until now, mutations in or deletions of *SST* have not been associated with tall stature in the medical literature. There exists, however, evidence that this gene is likely to cause alterations in height. First, models show that mutations in the *SST* gene are associated with increased body weight, length, and circumference in different animals (26, 27). Somatostatin analogs have also been used to successfully reduce IGF-1 and GH levels (and ultimately final height) in tall patients (48, 49). If somatostatin analogs can reduce final height, it is possible that reducing somatostatin levels through a mutation or deletion of *SST* can lead to increased height and tall stature. Finally, in addition to our patient, there were four other descriptions in the DECIPHER database of patients with deletions of chromosome 3 that overlapped with *SST* whose described phenotypes included tall stature (Supplementary Table 1). One patient (DECIPHER ID 260893) had only two haploinsufficient protein-coding genes affected in their CNV deletion, *SST* and *BCL*6. BCL6 is a transcriptional repressor important in germinal center formation, and dysregulation of *BCL6* is associated with diffuse large cell lymphoma and follicular lymphoma (50). Given the strong physiological association between SST and GH, *SST* is a stronger candidate gene than *BCL6*, with a biologically feasible mechanism of action.

In summary, through ES analysis, we were able to achieve genetic diagnosis for 11 out of 37 patients with syndromic tall stature, resulting in a diagnostic yield of 29.7%. This study was limited by a relatively small number of patients, some of whom have already been reported in prior studies, as listed above. Another limitation of this study was that not all genes identified in this paper were able to be segregated via Sanger sequencing to confirm inheritance. Our study uncovered four novel candidate genes for syndromic tall stature, motivating future work to continue clarifying the effects of these candidate genes on stature, and, more generally, to continue identifying rare, monogenic causes of syndromic tall stature so that specific treatment and management recommendations can be developed for these patients.

## Supplementary materials



## Declaration of interest

AALJ has received consulting fees from Novo Nordisk and has an independent research grant from BioMarin. The other authors declare that they have no competing financial interests.

## Funding

This work was supported by Grants 2013/08028-1, 2022/10107-6, and 2023/09879-7 from the São Paulo Research Foundationhttps://doi.org/10.13039/501100001807 (FAPESP); Grant 303294/2020-5 from the National Council for Scientific and Technological Developmenthttps://doi.org/10.13039/501100003593 (CNPq); and by Coordination of Superior Level Staff Improvement (CAPES; Finance Code 001 to RCR and LPC).

## Ethics statement

This study was approved by the Research Ethics Committee of the Hospital das Clínicas at the Universidade de São Paulo and conducted per the principles of the Declaration of Helsinki. Patients and/or their parents or legal guardians provided formal consent for clinical and genetic analysis.
