# Supplementary material for: Exome sequencing of patients with syndromic tall stature reveals four novel candidate genes
Source: Endocr Connect. 2025 Jul 15;14(7):e250137. doi: 10.1530/EC-25-0137 (PMC12268985; doi:10.1530/EC-25-0137)
Supplement: Supplementary file 1 [file supplementary_materials.pdf]

Supplementary Table 1. Patients with overlapping CNVs and/or point mutations listed in the DECIPHER database.

Patient	Gene	Variant [GRCh38]	Variant Size (kb)	Protein Coding Genes	Genes displaying Haploinsufficiency	Clinical Features
**ID 13**	*PTCH1*	DEL:chr9-94443367-96223323	1,779,956	7	*PTCH1*	Abnormality of the vertebral spinous processes, basal cell carcinoma, concave nasal ridge, genu valgum, hypertrichosis, jaw neoplasm, prominent forehead, tall stature, twelfth rib hypoplasia
**Decipher ID 290013**	*PTCH1*	DEL:chr9-95504799-95510094	5,295	1	*PTCH1*	Slender build, specific learning disability, tall stature
**Decipher ID 761**	*PTCH1*	DEL:chr9-94573763-99608115	5,03.4,352	37	*PTCH1, NCBP1, GABBR2, ANP32B, TGFBR1, FOXE1, TMOD1, COL15A1*	Delayed speech and language development, intellectual disability, tall stature
**Decipher ID 249750**	*PTCH1*	DEL:chr9-93446463-101838687	8,392,224	63	*PTCH1, FAM120A, NCBP1, TEX10, GABBR2, ANP32B, NR4A3, TGFBR1, PHF2, FOXE1, ZNF367, TMOD1, ERP44, COL15A1*	Brachycephaly, epicanthus, large hands, long eyelashes, long foot, microdontia, short neck, submucous cleft hard palate, tall stature, wide intermamillary distance, wide nasal bridge
**Decipher ID 276843**	*PTCH1*	chr9-95469155T>C (c.1848-2A>G)	-	1	*PTCH1*	Asymmetric growth, bifid ribs, bifid and broad uvula, delayed eruption of permanent teeth, epicanthus, high palate, pectus excavatum, relative macrocephaly, tall stature, webbed neck
**ID 14**	*SST*	DEL:chr3-187125493-190748454	3,622,961	15	*TP63, BCL6, CLDN1, SST, IL1RAP, TPRG1, MASP1*	Abnormal earlobe morphology, absence seizures, esophagitis, gastritis (mild), mandibular prognathia, splenomegaly
**Decipher ID 258378**	*SST*	DEL:chr3-187460994-187874608	413,614	3	*BCL6, SST*	Autism, avascular necrosis of the capital femoral epiphysis, focal aware seizure, intellectual disability, tall stature

**Decipher ID 260893**	*SST*	DEL:chr3-185921110-189997974	4,076,864	25	*TP63*, *ETV5*, *TRA2B*, *BCL6*, *EIF4A2*, *ST6GAL1*, *DNAJB11*, *SST*, *TPRG1*, *MASP1*	Arachnodactyly, disproportionate tall stature, downslanting palpebral fissures, feeding difficulties in infancy, high palate, intellectual disability, long toe, low hanging columella, malabsorption, pectus carinatum and excavatum, posteriorly rotated ears, prominent nasal bridge, scoliosis, slender build
**Decipher ID 277546**	*SST*	DEL:chr3-184518947-190803820	6,284,873	39	*TP63*, *ETV5*, *TRA2B*, *BCL6*, *IGF2BP2*, *EIF4A2*, *MAP3K13*, *ST6GAL1*, *DNAJB11*, *CLDN1*, *SENP2*, *EPHB3*, *SST*, *IL1RAP*, *TPRG1*, *LIPH*, *MASP1*	Deeply set eye, disproportionate tall stature, high palate, hypodontia, intellectual disability (moderate), long face, memory impairment, osteopenia, pes planus, impaired social interactions
**Decipher ID 308182**	*SST*	DEL:chr3-187460995-192102091	4,641,096	16	*TP63*, *BCL6*, *CLDN1*, *GMNC*, *SST*, *IL1RAP*, *TPRG1*, *CCDC50*	Constipation, dental crowding, low-set ears, macrocephaly, sandal gap, seizure, tall stature

Information from DECIPHER drawn from (https://decipher. sanger.ac.uk/ application) (22).
